# DoDELLA-GAI2 Integrates Gibberellin and Ethylene Signaling to Regulate Chinese Yam (*Dioscorea opposita*) Tuber Development

**DOI:** 10.3390/biology14060635

**Published:** 2025-05-30

**Authors:** Mingran Ge, Yanfang Zhang, Yanping Xing, Linan Xing, Huiqin Miao, Xiuwen Huo

**Affiliations:** 1 Horticulture Department, Faculty of Horticulture and Plant Protection Science, Inner Mongolia Agricultural University, Hohhot 010019, China; gemingran1996@163.com (M.G.); zhangyanfang@imau.edu.cn (Y.Z.); xln620719@163.com (L.X.); 2 Biochemistry and Molecular Biology, School of Life Sciences, Inner Mongolia Agricultural University, Hohhot 010019, China; xyping8315@163.com; 3 Library, Inner Mongolia Agricultural University, Hohhot 010019, China; miaohuiqin_2010@163.com

**Keywords:** *Dioscorea opposita*, gibberellin, *DELLA-GAI*, signaling cascade reaction, interaction mechanism

## Abstract

Yam (*Dioscorea opposita*) tuber development is a complex process regulated by various phytohormones, with gibberellin (GA) playing a crucial role. However, the underlying mechanisms and interaction of GA with other phytohormone pathways on yam tuber development remain incompletely understood. This study investigated the regulatory role of GA and its crosstalk with other phytohormones during yam tuber growth through phenotypic, cytological, physiological, and transcriptomic as well as targeted phytohormone metabolomics analyses. The results reveal that exogenous GA promoted tuber enlargement increases vascular bundle and the number and diameter of sieve tubes, and alters the expression of GA anabolism genes and GA signal transduction pathways. Integrated transcriptome and targeted metabolomics analyses revealed coordinated changes in GA and ethylene (ETH) biosynthesis and signaling pathways during tuber development, particularly *DELLA-GAI2* acting as a negative regulator of GA signaling. Overexpression of *DoDELLA-GAI2* in transgenic tobacco significantly reduced GA level, starch, cytokinin (CTK), and ETH content, as well as aerenchyma tissue growth and parenchyma cell size. Exogenous GA and ethephon treatments increased GA, starch, CTK, and ETH content, and downregulated *DoDELLA-GAI2* gene expression. The yeast two-hybrid and bimolecular fluorescence complementation (BiFC) assays confirmed a direct interaction between *DoDELLA-GAI2* and *DoMTCPB*, an upstream gene-encoding key enzyme in ETH biosynthesis. *DoDELLA-GAI2* acts as a negative regulator of ETH synthesis by interacting with *DoMTCPB*. GA-induced degradation of *DoDELLA*-*GAI2* relieves this inhibition, promoting ETH production and contributing to tuber growth. Taken together, our findings reveal a novel mechanism based on *DoDELLA-GAI2* integrating the GA and ETH signaling processes to regulate tuber development in *D. opposita*, offering a potential target for improving yam crop productivity.

## Simple Summary

Yam is a vital food crop, but fully understanding how its edible tubers grow is key to improving harvests. Plant growth is controlled by natural signals called phytohormones. We investigated how two important phytohormones, gibberellin (which encourages growth) and ethylene (involved in various growth processes), work together in yam tubers. Our goal was to uncover the molecular link connecting these two signals. We found that applying gibberellin made tubers larger and changed phytohormone levels. We discovered a specific protein, DoDELLA-GAI2, which normally acts as a brake on gibberellin’s action, directly interacts with another protein (DoMTCPB) involved in making ethylene. When gibberellin levels are high, the DoDELLA-GAI2 brake is released, allowing the ethylene-making protein to become more active, leading to more ethylene production, which helps the tuber expand. This study reveals how the DoDELLA-GAI2 protein acts like a control switch linking gibberellin and ethylene signals to regulate yam tuber size. Understanding this natural control mechanism can lead to new strategies for growing yams more efficiently or increasing tuber size, ultimately benefiting food production and agriculture.

## 1. Introduction

Yam (*Dioscorea opposita*), belonging to the Dioscoreaceae family, is a globally important tuber crop with significant nutritional and economic value [1,2]. The yam tuber, known as “Chinese little ginseng” in China, has a long history of use as a medicinal and edible material. In East Asia, Chinese yam (*Dioscorea polystachya* Turcz.) is cultivated as a staple vegetable, whereas several yam species are used as staple foods in West Africa [1,2]. Yam tubers, depending on the specific variety, contain starch (~65%), protein (~9%), and fiber (~1.2%) [3], and are rich in minerals beneficial bioactive compounds, including allantoin and diosgenin, making it useful for treating various diseases, including asthma, diarrhea, and diabetes [3,4]. Given its agricultural value as a food crop and its medicinal properties, understanding the mechanisms that regulate yam tuber growth holds considerable importance for improving its yield.

Yam tuber formation is a complex process that involves three phases: initiation, expansion, and commercial maturation stages [5]. Tubers are formed from the swelling of underground stems characterized by a large accumulation of starch and storage proteins, and the expansion of parenchyma cells. This process is influenced by genotype, the environment, and various factors, including enzymes, phytohormones, and their downstream signaling pathways [6]. Endogenous phytohormones, including gibberellin (GA), auxin (IAA), and abscisic acid (ABA), are crucial for the initiation stage of tuber, with cytokinin (CTK), ethylene (ETH), and jasmonic acid (JA) participating in tuber development and growth [7]. ABA has been shown to positively regulate tuber formation in yam, and ETH is also involved in the regulation of microtuber formation, with multiple ethylene-related genes upregulated during early tuber development [8]. While over 100 types of GAs have been discovered, of which only GA1, GA3, GA4, and GA7 exhibit bioactivities, the rest are metabolites or intermediates [9]. GAs are critical regulators of plant development and play well-known roles in increasing crop yields. Relevant to overall plant vigor that supports tuber development, GAs promote cell division and elongation [10], increase lodging resistance [11], can influence the flowering period and endogenous phytohormone balance [12], and promote photosynthesis to increase carbohydrate production for underground parts [13].

Previous research has demonstrated that a high level of endogenous GA is produced in the early period of yam tuber enlargement [14], and the GA content is significantly correlated with starch synthesis [15]. Moreover, exogenous GA application has been proven to promote yam tuber growth and development [16], and subsequent work established a mechanism involving GA3 regulation via a DELLA-dependent pathway influencing GA metabolism, signaling, and cell wall-related genes [17]. However, phytohormones do not function independently, but rather crosstalk with each other. For instance, in potato (*Solanum tuberosum*), GA-ETH crosstalk regulates sprouting via DELLA protein degradation [17], while in cassava (*Manihot esculenta*), phytohormone crosstalk involving auxin, cytokinin, and other phytohormones regulate tuberous root development [18]. In yam, the specific molecular mechanism of such phytohormone crosstalk is not clear. Particularly, the precise molecular mechanisms by which GA regulates yam tuber development, and the complex interactions with other phytohormones, such as ETH, require further elucidation. Therefore, it is speculated that further investigation into the downstream components of GA signaling and their interactions with other phytohormone signaling pathways are needed to fully understand the regulation of yam tuber development.

This research investigated how GA signaling interacts with other plant hormone pathways to regulate yam tuber enlargement. We hypothesized that when exposed to varying levels of GA, as well as the GA biosynthesis inhibitor (such as paclobutrazol, PAC), the growth of yam tubers would be affected. Through a transcriptional analysis of genes related to plant hormone metabolism and signaling pathways in yam, we identified the key regulatory gene *DELLA-GAI2* of the GA pathway and verified the functions to determine upstream and downstream regulatory effects. This study aims to provide deeper insights into the GA-mediated development of yam tubers.

## 2. Materials and Methods

### 2.1. Plants, Cultivation, and Treatments

Chinese yam (*Dioscorea opposita* Thunb.) cultivar ‘Dahechangyu’ (DHCY) was planted at the yam germplasm resource nursery, located at Inner Mongolia Agricultural University, Hohhot, China (E 110°46′–112°10′ and N 45°51′–41°8′) in July 2023. The climate at the planting site is classified as temperate, continental monsoon. Yam tuber growth period was divided into three periods: the initiation stage (90–105 days after planting, DAP), enlargement stage (105–135 DAP), and commercial maturation stage (135–165 DAP).

At the beginning of the tuber initiation stage (90 DAP), plants were randomly subjected to the following treatments (150 plants per treatment group): control (Con): sprayed with water, GA-treated plants were sprayed with 200 mg/L GA (GA3), and PAC-treated plants were sprayed with 200 mg/L GA biosynthesis inhibitor (Paclobutrazol, PAC, Sigma-Aldrich, St. Louis, MO, USA). The solutions were sprayed onto the leaf surfaces, first treatment was applied at the initiation stage (90 DAP), and the second treatment was applied 7 days later (97 DAP). For all treatments, tissue samples (tubers, stems, and young leaves) were collected (every 15 DAP, for a total of 5 different periods) at 105, 120, 135, 150, and 165 DAP. At each sampling point and treatment, three different plants were randomly selected. For each biological replicate, the tissues were mixed together, and 3 replicates were processed. Samples were immediately frozen at −80 °C.

For targeted metabolomics analysis, samples were collected at three distinct developmental stages (with an interval of 30 days): 105, 135, and 165 DAP. For each time point, tubers from 6 plants were combined to form one biological replicate, with 6 replicates generated (*n* = 6). Samples were swiftly frozen at −80 °C and subsequently sent to NOA Zhiyuan Inc. (Beijing, China) for metabolomics sequencing.

### 2.2. Morphological, Physiological, and Histological Analyses

Tuber length and diameter of each sample were measured using a ruler and Vernier caliper. After washing, the fresh tubers were weighed using an electronic scale. Starch content was quantified via the iodine absorbance approach [19], while reducing sugar levels were assayed using the 3,5-dinitrosalicylic acid method [20,21]. GA concentrations were analyzed using an ELISA kit (Sino Biological, Shanghai, China). Enzyme activities of sucrose phosphate synthase (SPS) and soluble starch synthase (SSS) were determined using the colorimetric method [22,23].

For paraffin sectioning, fresh yam tubers were cross-sectioned and cut into 1/4 pieces and placed in formaldehyde–acetate–ethanol fixative (FFA). The fixed samples were subjected to standard paraffin, sectioned using a microtome, stained using Fast Green, and visualized via a confocal microscope (C2-ER; Nikon, Tokyo, Japan).

### 2.3. Key Gene Expression Patterns Analysis

Total RNA was isolated from GA- and PAC-treated tubers for first-strand cDNA synthesis. Expression of genes critical to the GA pathway, including DoDELLA, DoGID1, DoGID2, DoKS, DoKAO, DoGA20ox, DoGA2ox, DoGA3ox, and DoMTCPB, were analyzed using qRT-PCR according to a previously reported method [24]. Yam ubiquitin (UBQ) gene served as an endogenous control. Primers designed using Primer Premier 5.0 (Premier Biosoft, Palo Alto, CA, USA) are shown in Table S1.

### 2.4. Targeted Metabolomics Analysis

Metabolomics analysis was performed on tuber samples from the three rapid growth stages collected at 105, 135, and 165 DAP. Phytohormones were quantitated using ultra-high-performance liquid chromatography-tandem mass spectrometry (UHPLC-MS/MS) (Novogene, Beijing, China) [25]. Metabolite identification and annotation were conducted using the Kyoto Encyclopedia of Genes and Genomes (KEGG) database, and the raw data were processed with MetaX (version 1.4.16) [26]. Principal component analysis (PCA) and orthogonal partial least squares discriminant analysis (OPLS-DA) were applied to evaluate the metabolic profiles. Differential accumulation metabolites (DAMs) were identified using thresholds of variable importance in projection (VIP) ≥ 1, fold change (FC) > 1.2 or FC < 0.5, and statistical significance (*p* < 0.05) [27].

To analyze the relationship between gene expression and metabolomic data, KEGG pathway analysis was performed using differentially expressed genes (DEGs) and DAMs to identify shared pathways between DEGs and DAMs [28,29]. A correlation network diagram was generated to elucidate the functional interactions between DAMs and DEGs of phytohormone signal transduction pathways in the plants.

### 2.5. Isolation and Cloning of the DoDELLA-GAI2 Gene

The *DoDELLA-GAI2* gene (transcript_HQ_D_transcript22376/f2p0/1823) was screened based on transcriptome and metabolome data. Open reading frame (ORF) sequences of *DoDELLA-GAI2* were amplified using primers DoDELLA-GAI2-ORF-F/R. PCR was conducted using Ex Taq™ DNA polymerase (TaKaRa, Dalian, China), and the reaction system and procedures were operated according to its instructions, and subsequently sequenced via commercial services (Sangon Biotech, Shanghai, China).

### 2.6. Subcellular Localization and Functional Validation of the DoDELLA-GAI2 Gene

*DoDELLA-GAI2* coding sequence (CDSs), with the stop codon excluded, were fused to the CaMV35S-GFP vector for subcellular location determination [30]. Recombinant plasmids were subsequently transformed into the *Agrobacterium* tumefaciens strain EHA4404 via the freeze-thaw transformation [31]. The resulting bacterial culture was used to infiltrate *Nicotiana tabacum* L. (tobacco) leaves [32]. Confocal microscopy (C2-ER; Nikon, Tokyo, Japan) was employed to detect the green fluorescent protein (GFP) signal in the leaf epidermal cells.

For functional validation, the pPZP221-DoDELLA-GAI2 expression vector was generated and subsequently introduced into *Agrobacterium* tumefaciens strain EHA101. Wild-type (WT) tobacco plants were treated with the recombinant bacteria, and the transgenic lines were screened on 1/2 MS medium adding gentamicin (50 mg/L) and cefotaxim (250 mg/L) [33]. DNA and total RNA of the transgenic *DoDELLA-GAI2* and WT plants were isolated using a commercial plant extraction kit (CWBIO, Beijing, China). Three independent transgenic lines (*DoDELLA-GAI2-4*, *DoDELLA-GAI2-5*, and *DoDELLA-GAI2-8*) were selected for further analysis.

T0 generation and WT tobacco seeds were sown in the substrate and grown for approximately 1 month. WT tobacco was used as a control to observe the anatomical structures of stems and roots using a microscope (C2-ER; Nikon, Tokyo, Japan). Uniformly developed plants were selected and treated with GA (200 mg/L) and ethephon (200 mg/L), respectively (3 plants per treatment group), and the data were determined based on our preliminary trials. Samples were collected at 0, 12, and 24 h after treatment. Physiological indicators, including the expression of the *DoDELLA-GAI2* gene and starch content, were determined. The contents of gibberellin (GA), auxin (IAA), cytokinin (CTK), and ethylene (ETH) were extracted and measured using an assay kit (Bestlink Biotech, Shanghai, China).

### 2.7. Yeast Two-Hybrid (Y2H) Assay

To perform the Y2H assay, the bait vector pGBKT7-DoDELLA-GAI2 was constructed, and DoDELLA-GAI2-SZ-F/R primers containing *Sal I* and *Pst I* enzyme restriction sites (TaKaRa, Dalian, China) were designed (Table S1) to amplify the ORF sequence of the *DoDELLA-GAI2* gene. The combinations of pGADT7-largeT and pGBKT7-p53 (positive control), pGADT7-largeT and pGBKT7-laminC (negative control), and pGADT7 and pGBKT7-DoDELLA-GAI2 (test group) were transformed into AH109 yeast competent cells (Coolaber Inc., Beijing, China) [34,35]. Then, they were resuspended in ddH_2_O and spread on SD/-Trp/-Leu, SD/-His/-Trp/-Leu, and SD/-His/-Trp/-Leu/+Xa-Gal culture media (Coolaber Inc., Beijing, China), and incubated at 30 °C for 3 days. Based on the growth of colonies and the formation of blue plaques, the transcriptional autoactivation activity of the bait vector was detected.

The secondary yeast library plasmid of 20 µg was transformed into the bait yeast strain pGBKT7-DoDELLA-GAI2-AH109-competent cells [35]. The transformed resuspended bacterial liquid of 150 µL was spread on the SD/-His/-Trp/-Leu culture media and cultured at 30 °C for 3–5 days. PCR identification was performed using 5′AD/3′AD and subjected to sequencing at Sangon Biotech (Shanghai, China). Blast analysis (NCBI, https://www.ncbi.nlm.nih.gov accessed on 2 March 2024) was used to identify potential interacting proteins.

For Y2H assays, *DoMTCPB* and *DoDEX1* CDS were inserted into pGADT7 at the *BamH I*/*Xhol* sites (TaKaRa, Dalian, China) to generate DoMTCPB-pGADT7 and DoDEX1-pGADT7. The resulting bait and prey vectors were co-transformed into strain AH109, spread on SD/-Trp/-Leu and SD/-His/-Trp/-Leu/+Xa-Gal culture media, and incubated at 30 °C for 3 days. The transcriptional autoactivation activity of the bait vector was determined based on the formation of colonies and the development of blue plaques.

### 2.8. Bimolecular Fluorescence Complementation (BiFC) Assays

CDS of *DoDELLA-GAI2* was cloned into the pBiFC-VC155 vector via the *KpnI*/*Xhol* site to generate nYFP-DoDELLA-GAI2, whereas *DoMTCPB* and *DoDEX1* CDS were inserted into the pBiFC-VN155 vector at the *Kpn I* and *Xhol* sites (TaKaRa, Dalian, China) to generate DoMTCPB-cYFP and DoDEX1-cYFP. All vectors were introduced into GV3101 and expressed in tobacco leaves [36], to observe fluorescence using a laser confocal microscope.

### 2.9. Statistical Analyses

Data were reported as the means ± standard deviations (SDs), and data were obtained from a minimum of three biological replicates. Statistical analyses were performed using SPSS V26.0 (IBM Corp., Armonk, NY, USA). Statistical significance was determined using one-way analysis of variance (ANOVA) followed by Student’s *t*-test.

## 3. Results

### 3.1. Exogenous GA Enhances Yam Tubers’ Growth

**Figure 1:**
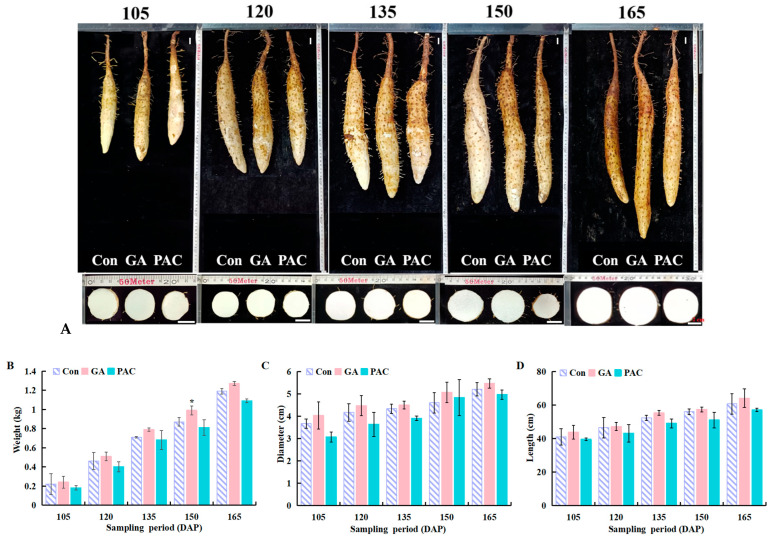

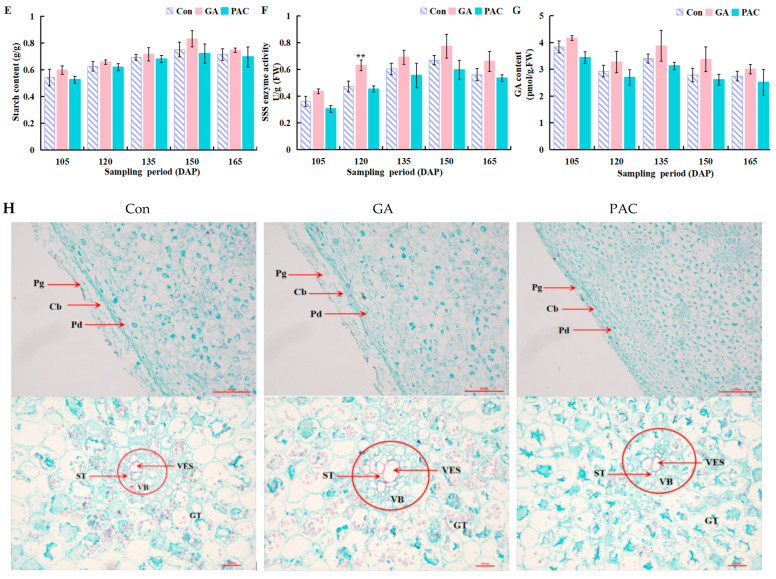
Exogenous GA treatment promotes tuber growth in yam. (**A**) Morphology of yam tubers at 105, 120, 135, 150, and 165 days after planting (DAP), treated with either water as a control (Con), 200 mg/L gibberellin (GA), or 200 mg/L paclobutrazol (PAC, GA biosynthesis inhibitor) (scale bars: 2 cm); (**B**) weight per plant; (**C**) tuber diameter; (**D**) tuber length; (**E**) starch content; (**F**) soluble starch synthase (SSS) enzyme activity; (**G**) gibberellin content; (**H**) cross-section of yam tubers at 105 DAP (scale bars: 100 μm), showing the basic tissue (GT), phellem layer (Pg), vascular bundle (VB), sieve tube (ST), vessel (VES), cork cambium (Cb), and phelloderm (Pd). Data in (**B**–**G**) are presented as the means ± SD (*n* = 3). *: *p* < 0.05, **: *p* < 0.01 compared to control, according to one-way ANOVA followed by Student’s *t*-test.

To examine GA’s physiological effects, yam tubers were treated with water as a control (Con), 200 mg/L GA, or 200 mg/L PAC at 90 DAP. During tuber expansion, exogenous GA application generally showed an overall increasing trend in indicators, including individual weight, diameter, length, starch content, SSS activity, and endogenous GA content, compared to the control (Figure 1B–G). Among these, significant differences were observed in tuber weight at 150 DAP and SSS activity at 120 DAP. Collectively, these observations suggest that exogenous GA treatment promotes tuber growth (Figure 1A–G).

Yam samples exposed to GA and PAC underwent paraffin-embedded sections for histological analysis (Figure 1H). The anatomical structure of the yam tuber was composed of periderm, basic tissue, and vascular bundle scattered in basic tissue. The periderm consists of the phellem layer, cork cambium and phelloderm. Inside the periderm was the basic tissue, which contained inclusions and starch granules. The tissue structure of the yam tuber was different after GA and PAC treatments (Table S2). GA-treated tubers had larger diameters and a higher number of vessels and sieve tubes compared to the control, whereas the PAC-treated tubers had lower values, which was consistent with the observed phenotypes. GA promoted tuber growth by promoting the number of vascular bundles, sieve tubes, and sieve tube diameters. The thickness of the cork cambium followed the order GA > control > PAC, and the thickness of the phelloderm in GA was remarkable higher in GA-treated tubers, compared to the PAC-treated tubers, demonstrating that GA affected the thickness of the cork cambium and phelloderm significantly.

### 3.2. GA Modulates the Expression of Key Genes in GA Metabolism and Signal Transduction Pathways

**Figure 2:**
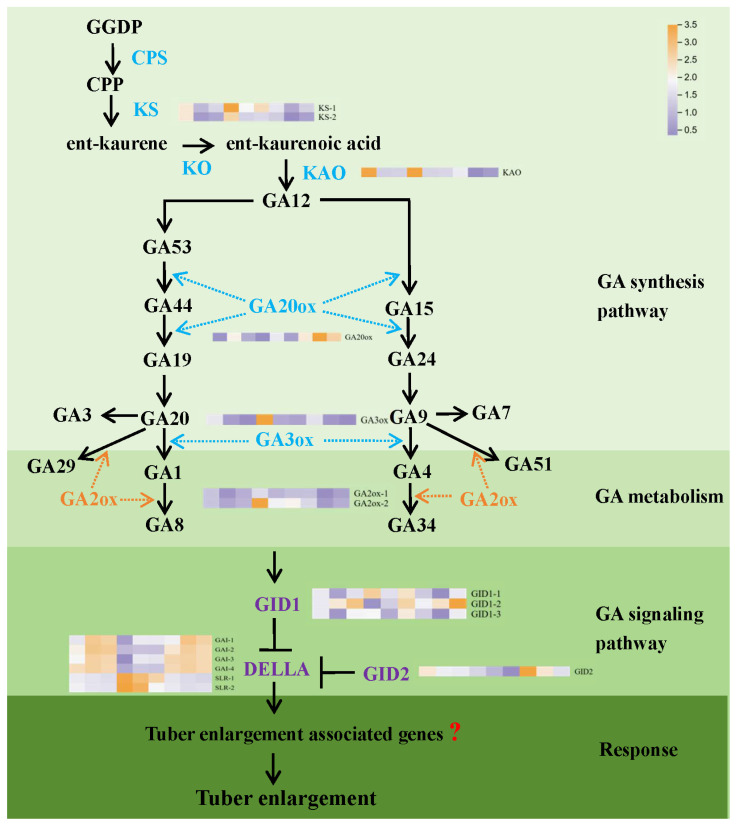
Effects of exogenous GA on key genes’ expression in GA anabolism and signal transduction pathways in tubers. Note: The heat maps (from left to right) represent gene expression levels in tubers at 105, 135, and 165 DAP, under Con (control), 200 mg/L GA (gibberellin), and 200 mg/L PAC (paclobutrazol) treatments (Con-105; Con-135; Con-165; GA-105; GA-135; GA-165; PAC-105; PAC-135; PAC-165). The colors (scale marked in the upper-right corner) indicate the log2 fold change in gene expression. GGDP: Geranylgeranyl diphosphate; CPS: Ent-copalyl diphosphate synthase; CPP: copalyl pyrophosphate; KS: Ent-kaurene synthase; KO: Ent-kaurene oxidase; KAO: Ent-kaurenoic acid oxidase; GA2ox: GA 2-oxidase; GA3ox: GA 3-oxidase; G20ox: GA 20-oxidase; GID: gibberellin-insensitive dwarf.

To explore the effects of GA and PAC treatments on GA biosynthesis and signal transduction genes, tuber samples at 105, 135, and 165 DAP were analyzed (Figure 2). During tuber development, *DoGID2* and *DoGA3ox* expression decreased, whereas *DoGID1*, *DoGA2ox*, *DoKS*, and *DoKAO* initially decreased but then increased. *DoGA20ox* and *DoDELLA* initially increased but then decreased. GA treatment increased the expression of the *DoGID1*, *DoGA3ox*, *DoGA2ox*, *DoKS*, and *DoKAO* genes, with *DoGA3ox* showing the most significant increase (by 1.88, 1.26, and 1.405 times, respectively). Conversely, GA treatment reduced the expression of the *DoDELLA*, *DoGID2*, and *DoGA20ox* genes, with *DoDELLA* showing the most significant reduction (by 58–76%, 25–68%, and 34–68%, respectively). PAC treatment showed the opposite effect. These results indicate that exogenous GA promotes tuber growth by affecting genes related to GA anabolism and signal transduction pathways.

### 3.3. Targeted Metabonomics Reveals Changes in Phytohormone Profiles During Tuber Development

**Figure 3:**
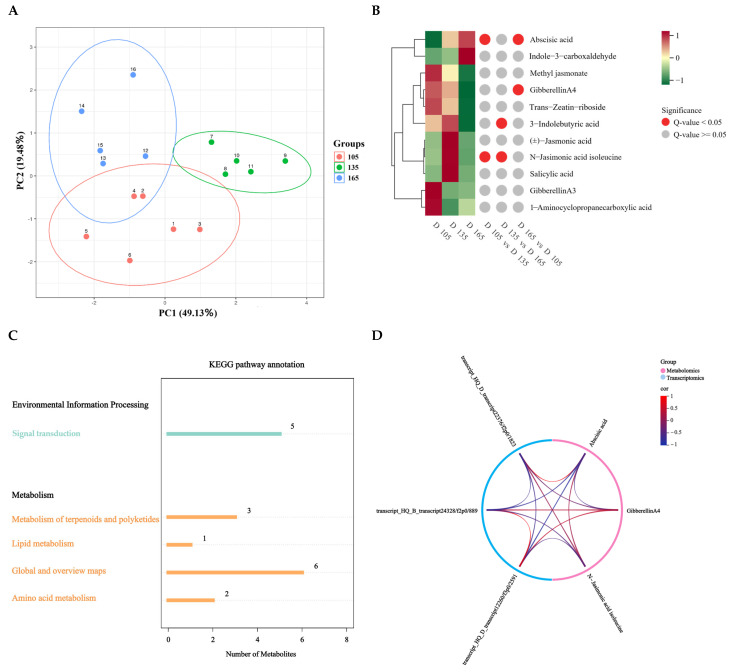
Targeted phytohormone metabonomics analysis of untreated yam during different developmental stages. (**A**) Principal component analysis (PCA) diagram (PC1, PC2: the first and second principal components; percentage of variance explained by each component). (**B**) Clustering heatmap of plant hormones. (**C**) Metabolite KEGG statistical diagram. (**D**) Correlation network of differentially expressed genes (DEGs) and differentially accumulated metabolites (DAMs) in plant hormone signaling pathways.

To further investigate the plant hormones in untreated yams at different developmental stages (at 105, 135, and 165 DAP), targeted phytohormone metabolomics analysis was performed using the LC-ESI-S/MS system. A total of 11 phytohormone metabolites were detected in all samples, including two GAs, two IAAs, three JAs, one CTK, one salicylic acid (SA), one ABA, and one ETH (Table S3). PCA results reveal that the samples were clearly separated based on different developmental stages, indicating good repeatability and reliability of the measurements (Figure 3A). OPLS-DA was used to further characterize the differences in phytohormone profiles, and to provide effective data for identifying the DAMs (Figure S1A–C).

DAMs were identified on the criteria of VIP ≥ 1, FC ≥ 1.2, FC ≤ 0.5, and *p*-value < 0.05. In the comparison between 105 vs. 135 DAP, JA-Ile and ABA showed a higher abundance. In the 135 vs. 165 DAP comparison, two DAMs, JA-Ile and IBA, showed a lower abundance. In the 165 vs. 105 DAP comparison, ABA showed a higher abundance, while GA4 showed a lower abundance. Clustering analysis showed that GA4 has the closest relationship with CTK, and GA3 has the closest relationship with ETH, which further suggests that GA may synergistically promote tuber growth with these two phytohormones (Figure 3B and Figure S1D–E).

KEGG pathway analysis was performed on the identified DEGs and DAMs to analyze the associations between genes and metabolites (Figure 3C and Table S4). Integration of transcriptomic and metabolomic data analysis revealed two co-enriched metabolite pathways that were simultaneously enriched with DEGs and DAMs, including signal transduction and terpenoids and polyketides metabolism. These pathways showed most DEGs and DAMs, indicating the crucial role of phytohormone signaling in the regulation of yam tuber development. The DEGs and DAMs within the phytohormone signal transduction pathways of yams were analyzed and the network diagram was constructed. GA4 is negatively regulated by transcript_HQ_D_transcript22376/f2p0/1823 (DELLA) and positively regulated by transcript_HQ_B_transcript 24328/f2p0/889 (AUX) and transcript_HQ_D_transcript 12260/f3p0/2591 (IAA) (Figure 3D). Therefore, we cloned transcript_HQ_D_transcript22376/f2p0/1823 and validated its function.

### 3.4. Dynamic Changes in Phytohormone Signal Transduction Pathways During Tuber Development

**Figure 4:**
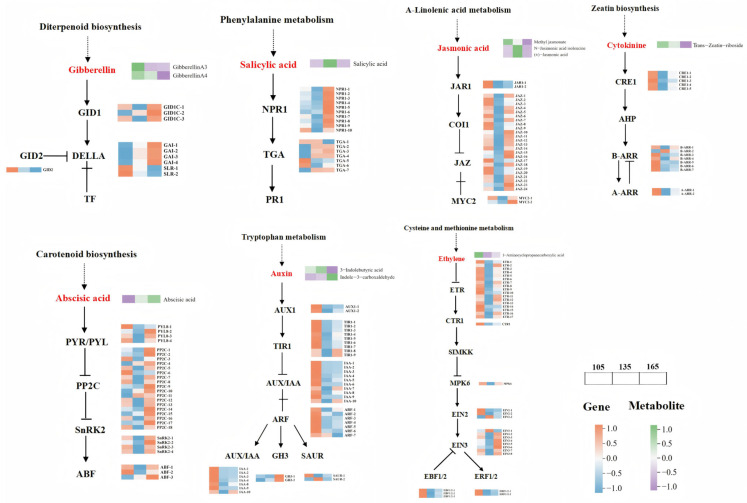
Dynamic changes in metabolites and genes in the plant hormone signal transduction pathway during yam tuber expansion. Note: The heatmap shows the metabolites and expression of genes at 105, 135, and 165 DAP (from left to right); the color change from orange to blue indicates gene expression from upregulation to downregulation; color change from green to purple indicates metabolites changing from higher relative abundance to lower relative abundance.

Dynamic changes in genes and metabolites involved in phytohormone signal transduction were observed during the tuber development process at 105, 135, and 165 DAP (Figure 4 and Table S5). In GA signaling, the expressions of *GID1* and *GAI1* were continuously upregulated. GA4, *GID2*, and *SLR* were highly expressed at 105 DAP, and then gradually decreased with tuber expansion. GA3 expression was first downregulated and then upregulated, with peak expression observed during the initiation stage of tuber expansion. In SA signaling, SA was elevated during the tuber enlargement stage, whereas *NPR1* and *TGA* expressions were continuously upregulated. In the CTK signaling pathway, the expressions of tZR, *CRE1*, *B-ARR*, and *A-ARR* were continuously downregulated. In the JA signaling pathway, *MYC2* was continuously upregulated, whereas MeJA and *JAR1* were continuously downregulated. *JAZ* was first downregulated and then upregulated, with a high expression at the initiation stage of tuber expansion. JA-Ile and JA were first upregulated and then downregulated. In the ABA pathway, ABA gradually increased, whereas *PYR/PYL*, *SnRK2*, *ABF*, and *PP2C* expression were first downregulated and then upregulated, reaching maximal expression during the commercial maturation stage. In the IAA pathway, ICA and *GH3* expressions were continuously upregulated, whereas *AUX1*, *TIR1*, *AUX/IAA*, *ARF*, and *SAUR* were highly expressed at 105 DAP, and then gradually decreased during tuber expansion. IBA was first upregulated and then slightly downregulated. In the ETH pathway, genes such as *ACC*, *ETR*, *CTR1*, *EBF1/2*, *MPK6*, *EIN2*, *EIN3*, and *ERF1/2* were first downregulated and then upregulated.

### 3.5. Isolation and Subcellular Localization of DoDELLA-GAI2

**Figure 5:**
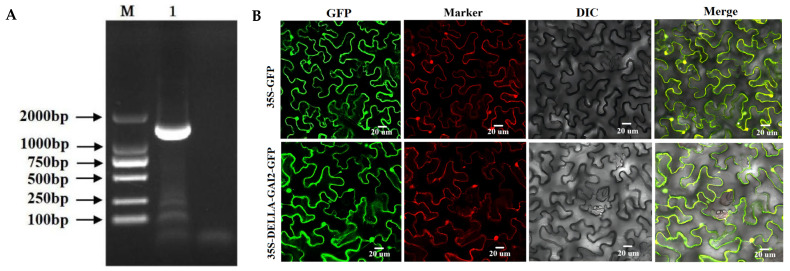
(**A**) Open reading frame (ORF) full-length PCR amplification of DoDELLA-GAI2 (M: DL2 000 DNA marker); for the original, uncropped, gel image, please refer to Supplementary Figure S2; (**B**) subcellular localization of DoDELLA-GAI2 fusion protein in tobacco leaves. Note: GFP: green fluorescent protein, marker: pBI221-NLS-CFP (nucleus) and pBI221-mCherry-PM (cytomembrane), DIC: Bright field, and Merge: Merged images (scale bars: 20 μm).

The ORF of *DoDELLA-GAI2* was obtained by PCR amplification, yielding a 1407 bp fragment (Figure 5A, for the original, uncropped, gel image, please refer to Supplementary Figure S2). The cDNA was designated as *DoDELLA-GAI2* (GenBank Accession No.: PP952097). Subcellular localization analysis using a DoDELLA-GAI2-GFP fusion vector showed that, compared with the GFP control protein, DoDELLA-GAI2 proteins were observed to be localized in both the cell membrane and nucleus (Figure 5B).

### 3.6. Overexpressed DoDELLA-GAI2 in Tobacco Affects Plant Growth and Anatomical Structure

**Figure 6:**
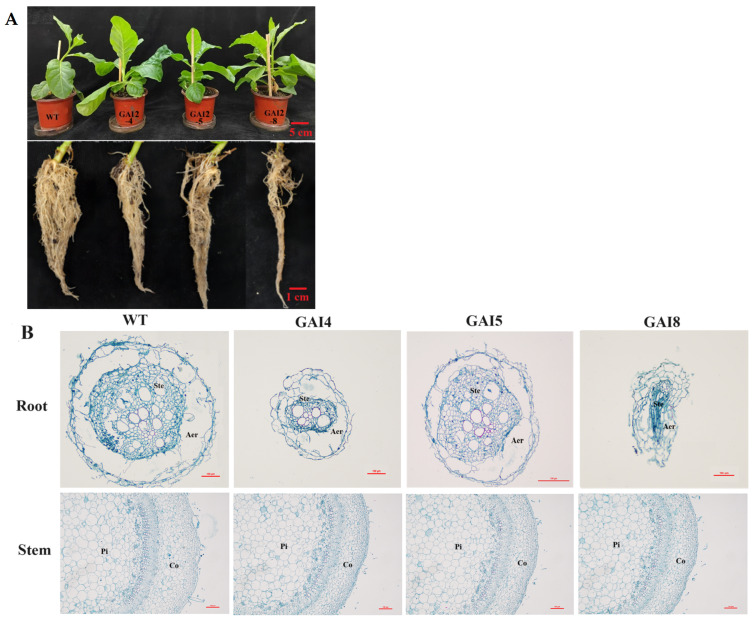

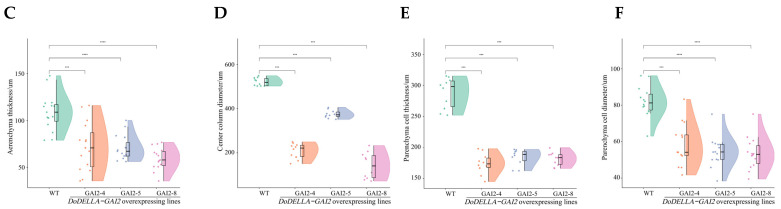
Analysis of the anatomical structure of overexpressed DoDELLA-GAI2 tobacco. Note: (**A**) plant growth and root phenotype (scale bar for plants: 5 cm; scale bar for roots: 1 cm); (**B**) cross-section of transgenic tobacco roots and stems (Aer: aerenchyma; Ste: stele; Pi: pith; Co: cortex; scale bars: 100 μm); (**C**) aerenchyma thickness; (**D**) center column diameter; (**E**) cortical parenchyma cell thickness; (**F**) parenchyma cell diameter. Data in (**C**–**F**) are presented as a distribution plot, with each point representing an individual measurement. ***: *p* < 0.001, ****: *p* < 0.0001.

To investigate the function of *DoDELLA-GAI2*, it was overexpressed in wild-type (WT) tobacco. Ten transgenic tobacco lines were obtained, and three T1 generation transgenic lines (GAI4, GAI5, and GAI8) were chosen for analyses. Compared with the control, the transgenic plants showed reduced growth of roots and stems, with significant decreases in aerenchyma thickness, central column diameter, parenchyma tissue thickness, and parenchyma cell diameter (Figure 6). These results indicate that *DoDELLA-GAI2* may negatively regulate plant growth by affecting the expansion of aerenchyma, vascular bundle, and parenchyma cell development.

### 3.7. GA Treatment Alters the Physiological Characteristics of Overexpressed DoDELLA-GAI2 Tobacco

**Figure 7:**
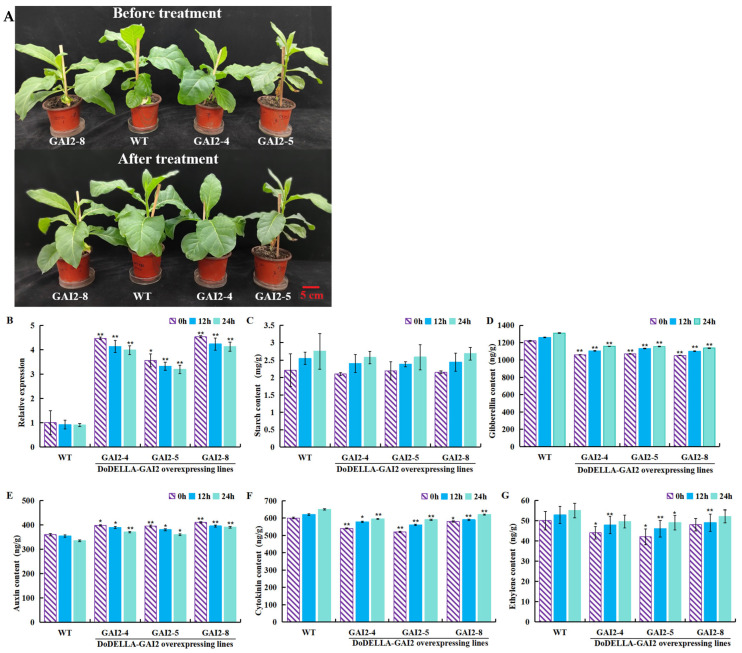
Changes in the physiology, biochemistry, and endogenous phytohormone content of *DoDELLA-GAI2* overexpressing tobacco under gibberellin (GA) treatment. (**A**) Plant growth (scale bar: 5 cm); (**B**) transcript levels of *DoDELLA-GAI2*; (**C**) starch content; (**D**) GA content; (**E**) auxin (IAA) content; (**F**) cytokinin (CTK) content; (**G**) ethylene (ETH) content. Note: Data are presented as mean ± SD (*n* = 3). *: *p* < 0.05, **: *p* < 0.01, compared to WT, according to one-way ANOVA followed by Student’s *t*-test.

To explore the reasons for phenotypic changes, the plants were sprayed with 200 mg/L GA. At 0, 12, and 24 h after treatment, changes in starch content, *DoDELLA-GAI2* expression, and endogenous phytohormone content were observed. Transgenic plants exhibited significantly higher *DoDELLA-GAI2* expression and higher IAA content, but lower contents of GA and CTK, compared to WT plants. After GA treatment, under different treatment times of the same lines, the contents of GA, starch, ETH, and CTK showed an increasing trend over time, reaching a peak at 24 h. Concurrently, *DoDELLA-GAI2* gene expression and IAA contents showed a decreasing trend with the increase in treatment time (Figure 7). These results show that *DoDELLA-GAI2* regulates plant growth by inhibiting the synthesis of GA.

### 3.8. Ethephon Treatment Alters Physiological Characteristics of Overexpressed DoDELLA-GAI2 Tobacco

**Figure 8:**
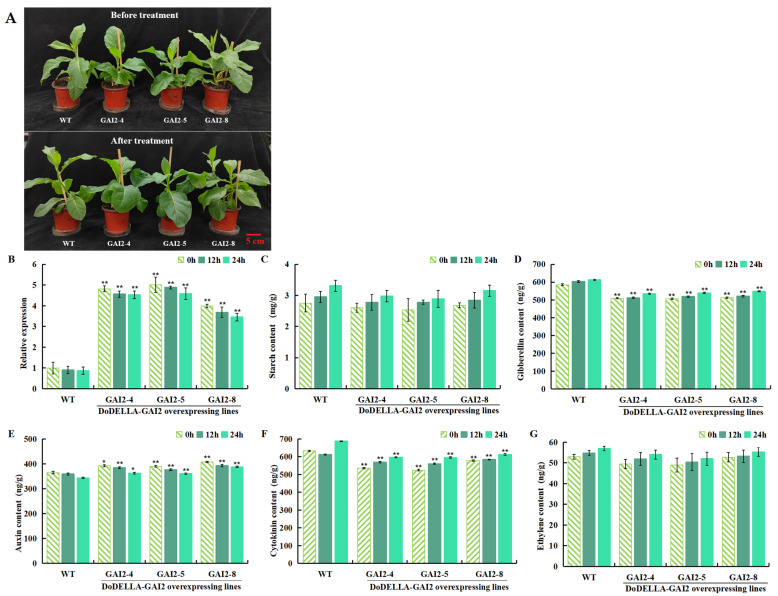
Changes in the physiology, biochemistry, and endogenous phytohormone content of DoDELLA-GAI2 overexpressing tobacco under ethephon treatment. (**A**) Plant growth (scale bar: 5 cm); (**B**) transcript levels of *DoDELLA-GAI2*; (**C**) starch content; (**D**) gibberellin (GA) content; (**E**) auxin (IAA) content; (**F**) cytokinin (CTK) content; (**G**) ETH content. Data are presented as mean ± SD (*n* = 3). *: *p* < 0.05, **: *p* < 0.01, compared to WT, according to one-way ANOVA followed by Student’s *t*-test.

To study whether ETH coordinated the regulation of plant growth through the GA signaling pathway, WT and transgenic tobacco plants were treated with 200 mg/L of ethephon. At 0, 12, and 24 h after treatment, changes in starch content, *DoDELLA-GAI2* gene expression, and endogenous phytohormone content were measured. Transgenic plants exhibited significantly higher *DoDELLA-GAI2* expression and IAA content, but lower contents of GA and CTK, compared to WT plants. After ethephon treatment, under different treatment times of the same lines, starch content, GA, CTK, and ETH content showed an increasing trend with treatment time, peaking at 24 h, whereas *DoDELLA-GAI2* gene expression and IAA content showed a decreasing trend (Figure 8). Ethephon treatment had a significant impact on transgenic plants, and GA may synergistically regulate plant growth and development by mediating the ETH pathway through *DoDELLA-GAI2*.

### 3.9. Self-Activation Detection of Decoy Vector pGBKT7-DoDELLA-GAI2

**Figure 9:**
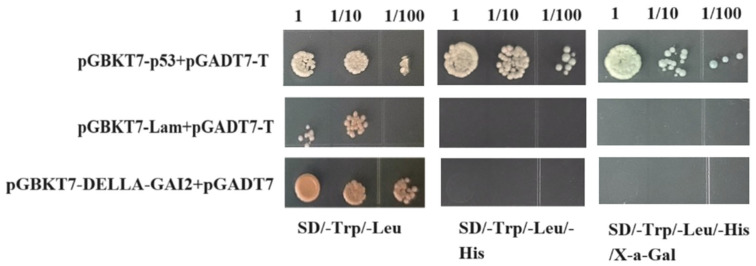
Toxicity detection of the bait vector pGBKT7-DoDELLA-GAI2. Note: Yeast cells expressing the pGBKT7-*DoDELLA-GAI2* bait vector with an empty pGADT7 vector, and the controls (positive: pGBKT7-p53 + pGADT7-T; negative: pGBKT7-laminC + pGADT7-T) were plated onto selective media. The numbers at the top indicate serial dilutions (1, 1/10, and 1/100). The positive control showed normal growth and a blue color on SD/-Trp/-Leu/-His/X-α-Gal media, whereas the bait vector showed no blue color and no growth on SD/-Trp/-Leu/-His/-Ade media, which indicates that the bait vector pGBKT7-*DoDELLA-GAI2* does not possess transcriptional autoactivation activity.

The pGBKT7-DoDELLA-GAI2 and pGADT7 vectors were co-transfected into *Saccharomyces cerevisiae* strain AH109 yeast-competent cells, to detect the autoactivation activity. The three bait vectors exhibited normal growth on SD/-Trp/-Leu, SD/-Trp/-Leu/-His/Xa-Gal, and SD/-Trp/-Leu/-His/-Ade media. Only the positive control (pGBKT7-p53 + pGADT7-T) displayed normal growth and appeared blue on X-a-Gal media (Figure 9), indicating that the bait plasmid pGBKT7-DoDELLA-GAI2 was successfully transferred into yeast cells and did not process self-activating activity; thus, it is suitable for subsequent library screening.

### 3.10. Screening and Identification of DoDELLA-GAI2 Interacting Proteins

**Figure 10:**
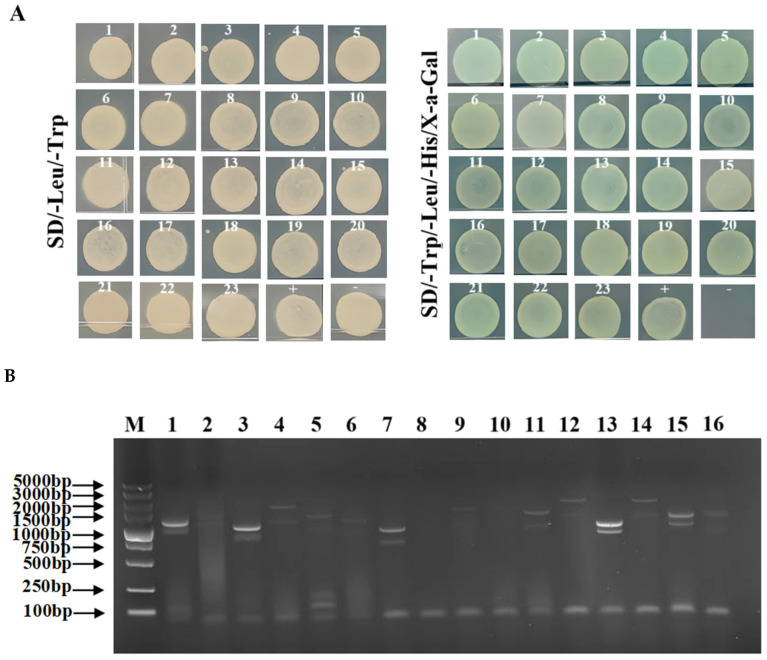
Screening of DoDELLA-GAI2 interacting proteins. (**A**) Colonies screened on SD/-Leu/-Trp and SD/-Leu/-Trp/-His/X-a-gal plates. 1–23: Numbering of screened positive clones; +: positive control; −: negative control. (**B**) representative colony PCR results for 16 clones. M: DL5000 DNA marker; 1–16: 1–16 bacterial solution PCR. The ID numbers in (**A**,**B**) and Table 1 are correspondingly consistent. For the original uncropped gel image, please refer to Supplementary Figure S3.

Yam cDNA library plasmid and pGBKT7-DoDELLA-GAI2 were co-transformed into yeast cells, and the resulting product was spread on SD/-Leu/-Trp/-His solid media and cultured. A total of 23 blue clones were obtained from the selective media on SD/-Leu/-Trp and SD/-Leu/-Trp/-His/X-a-gal plates (Figure 10A). The blue clones (SD/-Leu/-Trp/-His/X-a-gal plates) were selected for colony PCR, and the results are shown in Figure 10B (for the full Western blot, please refer to Supplementary Figure S3). The identified interacting proteins were confirmed by sequencing and BLAST (https://blast.ncbi.nlm.nih.gov/Blast.cgi, accessed on 2 March 2024) comparison, and a total of six interacting proteins were screened (Table 1). According to the gene function annotation results, these interacting proteins participate in multiple aspects of plant development, including photosynthesis, pollen development, signal transduction, and the abiotic stress response.

### 3.11. Yeast Two-Hybrid Point-to-Point Rotation Verification and Bimolecular Fluorescence Complementation Assays

**Figure 11:**
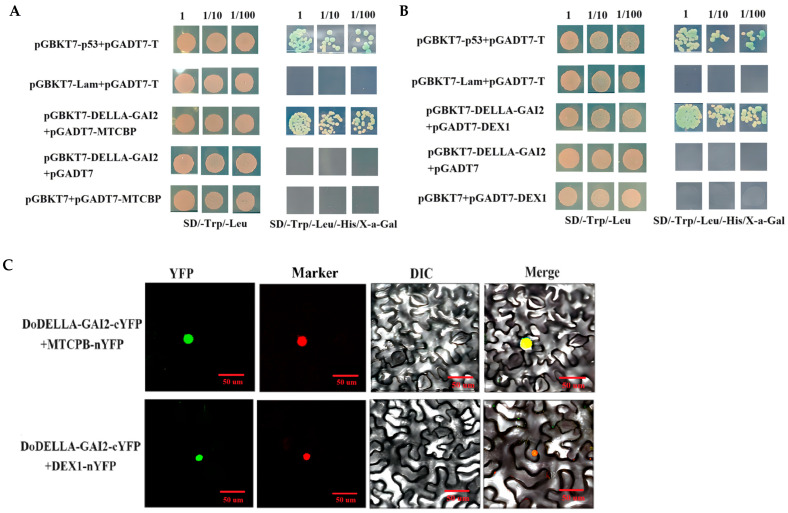
Yeast two-hybrid point-to-point rotation verification and BiFC confirmed the interaction of DoDELLA-GAI2 with DoMTCPB and DoDEX1 in tobacco cells. (**A**) DoDELLA-GAI2 and DoMTCPB rotation verification. (**B**) DoDELLA-GAI2 and DoDEX1 rotation verification. (**C**) DoDELLA-GAI2 and DoMTCPB. DoDELLA-GAI2 and DoDEX1. Note: YFP: YFP fluorescence, Marker: pBI221-NLS-CFP (nucleus), DIC: Bright field, Merge: Merged images (scale bars: 50 µm).

To investigate the interaction of *DoDELLA-GAI2* with potential candidate interacting proteins, the cDNA coding regions of the six candidates were cloned into the pGADT7 vector. Subsequently, point-to-point interaction analysis was conducted with pGBKT7-DoDELLA-GAI2. The results show that on SD/-Trp/-Leu/-HisX-α-Gal and SD/-Trp/-Leu selective media, only pGBKT7-DoDELLA-GAI2+ pGADT7-MTCPB/pGADT7-DEX1, along with the positive control (pGADT7-T + pGBKT7-p53) exhibits normal growth and blue colonies (Figure 11A,B). Moreover, the number of blue colonies decreased with increasing dilution factor, suggesting a specific interaction between DoDELLA-GAI2 and DoMTCPB as well as DoDEX1 in yeast.

As shown in Figure 11C and Figure S4, for the BiFC assay, green fluorescence was detected in *N. benthamiana* leaves co-expressing DoMTCPB-nYFP and DELLA-GAI2-cYFP, and DoDEX1-nYFP and DELLA-GAI2-cYFP. No signal was detected in DoMTCPB-nYFP, DoDEX1-nYFP + empty cYFP, or empty nYFP + DELLA-GAI2-cYFP. These findings confirm the direct interaction of DoMTCPB, DoDEX1, and DoDELLA-GAI2 in vivo.

### 3.12. Subcellular Localization of Interacting Proteins DoMTCPB and DoDEX1, and the Analysis of the Expression Pattern of DoMTCPB

**Figure 12:**
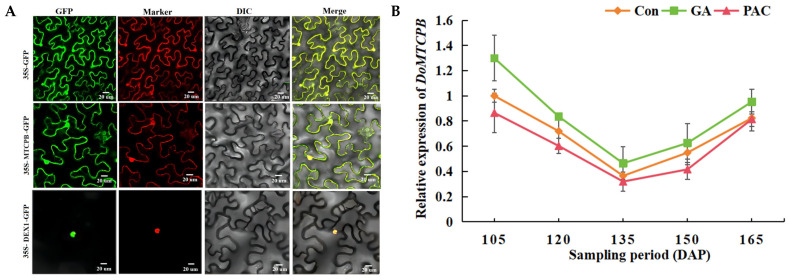
Subcellular localization of the interacting proteins DoMTCPB and DoDEX1, and expression pattern analysis of *DoMTCPB*. (**A**) Subcellular localization of DoMTCPB and DoDEX1 fusion proteins in tobacco leaves. GFP: green fluorescent protein; 35S-GFP and 35S-MTCPB-GFP markers: pBI221-NLS-CFP (nucleus) and pBI221-mCherry-PM (cytomembrane), 35S-DEX1-GFP marker: pBI221-NLS-CFP (nucleus); DIC: bright field; Merge: merged images; scale bars: 20 µm. (**B**) Expression pattern analysis of *DoMTCPB*. Data are presented as mean ± SD (*n* = 3).

To further understand the functions of interacting proteins DoMTCPB and DoDEX1 in the regulation of transcription, DoMTCPB-GFP and DoDEX1-GFP fusion vectors were constructed, with an empty GFP vector serving as a control. GFP control and DoMTCPB-GFP were localized in both the cell membrane and nucleus, whereas DoDEX1-GFP was localized in cell nucleus (Figure 12A).

DoMTCPB reached the highest level at 105 DAP, with the lowest expression at 135 DAP. Compared with the control, GA treatment increased DoMTCPB expression, whereas PAC treatment decreased it, indicating that DoMTCPB is responsive to GA stress (Figure 12B).

### 3.13. Integrated Model of GA and ETH Signaling During Tuber Development

**Figure 13:**
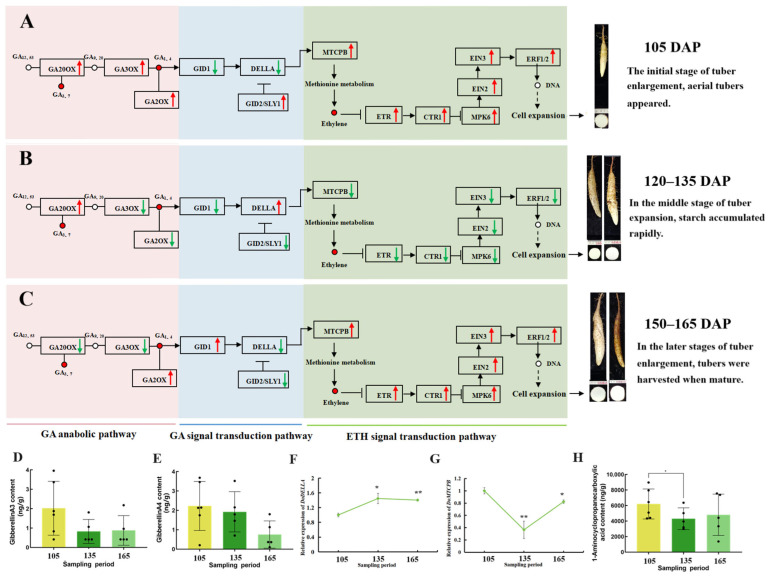
Regulatory mechanism of gibberellin (GA) and ethylene (ETH) signaling crosstalk during yam tuber development. (**A**–**C**) The integration of two KEGG pathways: diterpenoid biosynthesis (Ko00904) and plant hormone signal transduction (Ko04075), at three developmental stages: 105 DAP (days after planting, early stage), 120–135 DAP (middle stage), and 150–165 DAP (late stage). Black boxes: genes; circles: signaling intermediates; red arrows: high expression; green arrows: low expression. In the ETH pathway, ethylene binding to its receptor (ETR) leads to the inactivation of CTR1 (a negative regulator). This releases EIN2 from inhibition, allowing EIN2 to promote the stability and activity of EIN3/EIL1 transcription factors, which in turn activate ERF gene expression. (**D**) Gibberellin A3 (GA3) content. (**E**) Gibberellin A4 (GA4) content. (**F**) Relative expression of *DoDELLA-GAI2*. (**G**) Relative expression of DoMTCPB. (**H**) 1-aminocyclopropanecarboxylic acid (ACC) content. Data are presented as mean ± SD (*n* = 3). *: *p* < 0.05, **: *p* < 0.01 compared to 105 DAP, according to one-way ANOVA followed by Student’s *t*-test.

Based on the combined transcriptomic, metabolomic, and functional analyses, we propose a model illustrating the interplay between GA and ETH signaling during yam tuber development (Figure 13). In this model, GA promotes tuber growth, in part, by regulating ETH biosynthesis through the interaction between *DoDELLA-GAI2* and *DoMTCPB*. To determine this relationship, we analyzed the expression patterns of *DoDELLA-GAI2* and *DoMTCPB*, and measured the levels of GA3, GA4, and 1-aminocyclopropanecarboxylic acid (ACC) during tuber development.

The concentrations of GA3 and GA4 were the highest at 105 DAP, and then decreased significantly at later stages (135 and 165 DAP) (Figure 13D,E). Subsequently, *GA20ox* expression was high from 105 to 135 DAP but then decreased significantly at 150 and 165 DAP. *GA3ox* expression was high at 105 DAP, but decreased significantly from 120 to 165 DAP. The expression of *GA2ox*, which encodes a GA-deactivating enzyme, exhibited a different dynamic pattern: it was high at the initial stage (105 DAP), decreased significantly during the middle stages (120 and 135 DAP), and then increased again to high levels at the later stages (150 and 165 DAP) (Figure 13A–C). The high *GA20ox* and *GA3ox* expression at 105 DAP led to the GA3 and GA4 accumulations in tubers, which are necessary for the vegetative growth of yam tuber. On the contrary, at 165 DAP, reduced *GA20ox* and *GA3ox* expression, along with increased *GA2ox* expression in 165 DAP, corresponded with a significant decrease in GA3 and GA4 levels.

*DoDELLA-GAI2* expression was relatively low at 105 DAP, increased significantly at 120 and 135 DAP, and then decreased at 150 and 165 DAP (Figure 13F). *DoMTCPB* expression showed the opposite trend, with the highest expression occurring at 105 DAP and the lowest at 135 DAP (Figure 13G). ACC content, a precursor of ETH, showed a similar trend to that of GA3 and GA4 (Figure 13H). High GA3 and GA4 levels at 105 DAP likely promoted the formation of the GA-GID1-DELLA complex (Figure 13A–C), and the degradation of the DELLA protein led to a decrease in DELLA gene expression at 105 DAP. These changes release the expression of DoMTCPB and promote ethylene synthesis; the transcription causes the tuber to grow rapidly. DELLA protein degradation induced the expression of the DELLA gene to increase during 120 and 135 DAP, inhibiting the expression of DoMTCPB (Figure 13F–H).

These results support a model in which high GA levels at the early stage of tuber development (105 DAP) promote *DoDELLA-GAI2* degradation, releasing the inhibition of *DoMTCPB* and leading to increased ETH biosynthesis, which in turn facilitates tuber expansion. The dynamic changes in *DoDELLA-GAI2* and *DoMTCPB* expression, along with the corresponding changes in GA and ETH levels, suggest a finely tuned regulatory mechanism that controls yam tuber development.

## 4. Discussion

Improving crop yield and quality is a central goal of agricultural research. Yam is a globally significant tuber crop that ranks fourth in production after potato, cassava, and sweet potato [37]. Tuber development is regulated by the interaction of multiple factors, including genetic, environmental, and plant hormones factors. Previous studies have demonstrated that GAs influence tuber formation by enhancing sink strength, promoting nutrient translocation, and facilitating storage expansion [38]. In this study, we investigated the role of GA in tuber development and found that exogenous GA application at the early stage of tuber growth significantly increased tuber growth, starch accumulation, and the activity of SSS in yam tubers (Figure 1), consistent with the previous findings that GA promotes starch synthesis in tubers [39,40]. GA-regulated starch accumulation and SSS activity indicate that GA is vital for tuber development, possibly by affecting resource allocation and carbohydrate metabolism, as supported by a related study [41,42]. Furthermore, GA promotes the growth of vascular tissue, as evidenced by the enhanced elongation and division of sieve tubes (Figure 1). These findings indicate that GA enhances production of vascular tissues and the periderm [43], which is consistent with the findings of a similar study [44].

Plant hormone networks are highly complex, with multiple phytohormones influencing growth and development. By integrating targeted metabolomics and transcriptomics, we identified 11 key plant hormone metabolites, including 2 GAs 2 IAAs, 3 JAs, 1 CTK, 1 SA, 1 ABA and 1 ETH (Table S3), and found that GA3 and GA4 levels were closely associated with ETH and CTK, respectively (Figure 3B). These findings, combined with the expression patterns of DoGA20ox and DoGA3ox (Figure 2), indicate a potential co-regulation or crosstalk between these plant hormone pathways. Other studies have reported similar relationships between GA and other phytohormones. For example, GAs and cytokinins are known to antagonistically regulate each other’s levels in *Arabidopsis* [45]. The co-enrichment of DEGs and DAMs in the signal transduction and terpenoid/polyketide pathways further underscores the central role of phytohormone signaling in yam tuber development (Figure 3C and Table S4), which is consistent with the previous findings in yam [21]. Furthermore, correlation analysis of DEGs and DAMs in phytohormone signal transduction metabolic pathways showed that related DEGs play a positive or negative role in regulating DAMs, including the *DELLA-GAI2* of the GA signaling pathway, and *AUX22D* and *IAA17* of the auxin signal transduction pathway. These genes may cooperate with the signal transduction pathways of other plant hormones to promote tuber growth (Figure 3D), as shown in the previous findings [46]. GA biosynthetic and signaling pathways are well-established in model organisms, such as *Arabidopsis* and rice [47]. *GA20ox* and *GA3ox* are positive regulators of active GA biosynthesis, whereas *GA2ox* acts as a negative regulator [48]. Analysis of key GA biosynthesis genes in yam revealed that *GA20ox* and *GA3ox* expressions likely induce GA3 and GA4 biosynthesis in the early stages of yam tuber development. These findings indicate that GA biosynthesis is inhibited in the later stage of tuber growth, resulting in the conversion of bioactive GA3/GA4 into inactivated GA29/GA34 (Figure 13D–E).

The research demonstrated that the height and development of transgenic sweet potato plants are regulated through the ABA and GA signaling pathways [49]. GA-induced DELLA protein degradation is a central regulatory mechanism within GA signaling pathway [50], controlling GA synthesis, signal transduction and response, and the degradation of relevant genes [51]. To date, several DELLA proteins that belong to the GRAS/DELLA family have been identified in various plants [52]. Different members play key roles in plant development by integrating various phytohormone signals [53]. In our study, the overexpression of DoDELLA-GAI2 reduced the aerenchyma thickness, parenchyma cell size, and GA content of tobacco plants. These findings indicate that DELLA-GAI2 participates in plant growth by regulating GA biosynthesis (Figure 6 and Figure 7). DELLA proteins are known to interact with numerous partners, including transcription factors [54], thereby regulating the expression of downstream genes [55]. In rice, GA signaling promotes cellulose synthesis via the interaction of DELLA SLR1 and the transcription factor NACs for secondary-wall formation [56]. In *Arabidopsis thaliana*, the DELLA protein in the GA pathway and EIN3 in the ethylene pathway antagonistically regulate the expression of the phytochrome transcription factor PIF to regulate plant chlorophyll synthesis [57]. Therefore, elucidating the upstream and downstream signaling mechanisms of DELLA proteins is the key to better understanding how GAs regulate plant development. In this study, yeast two-hybrid and BiFC methods were used to screen out the interacting proteins of DoDELLA-GAI2, such as DEX1 and MTCPB. A key finding of this study is the direct physical interaction between DoDELLA-GAI2 and DoMTCPB, an enzyme in the methionine salvage pathway linked to ETH biosynthesis [58], as confirmed by Y2H and BiFC (Figure 11). After GA combines with GID1, the GA-GID1-DELLA complex is formed, which triggers DELLA protein degradation through the ubiquitin-proteasome pathway, and promotes the release of downstream signaling pathway response genes [59]. Our results show that spraying GA at the early stage of tuber expansion can increase the endogenous GA content (Figure 1G), leading to GA-GID1-DELLA complex formation. Subsequent DELLA degradation releases ETH signals, which promote the elongation and growth of the vascular tissue and cambial cells of the tuber, which is beneficial for the enlargement of the tuber. DoMTCPB expression was responsive to GA treatment (Figure 12B), further supporting its role in this crosstalk. Based on these findings, we propose that DoDELLA-GAI2 inhibits DoMTCPB upon GA perception, and that GID1 mediates DoDELLA-GAI2 degradation [59], releasing DoMTCPB to contribute to ETH synthesis. This model (Figure 13) aligns with the observed temporal patterns: high, early GA leads to low DoDELLA-GAI2 expression, high DoMTCPB expression, and high ACC levels, promoting initial tuber expansion.

However, this study has several limitations. The functional validation primarily relied on the heterologous tobacco system; however, these results require direct confirmation in yam itself, potentially through stable transformation or gene editing, which remains challenging in yam. Future work should aim to validate the DoDELLA-GAI2-DoMTCPB interaction and its consequences directly in yam tubers with larger sample sizes. Investigating the role of the other identified interactor, DoDEX1, could also provide further insights. Despite these limitations, this study provides the first evidence (to our knowledge) that a specific DELLA protein (DoDELLA-GAI2) directly interacts with an ETH biosynthesis-related enzyme (DoMTCPB) in the context of yam tuber development. This identified DoDELLA-GAI2-DoMTCPB module represents a crucial regulatory point integrating GA and ETH signals. Understanding such specific molecular interactions is essential for developing targeted strategies for crop improvement. Manipulating the stability of DoDELLA-GAI2 or the activity of DoMTCPB can offer novel avenues for modulating tuber initiation and growth rates in yam, potentially leading to increased yields.

## 5. Conclusions

This study elucidates a novel molecular mechanism by which GA and ETH were integrated into regular yam tuber development. We demonstrate that *DoDELLA-GAI2*, as a negative regulator of GA synthesis, directly interacts with *DoMTCPB*, a key enzyme in ETH biosynthesis, providing a mechanistic link between these two essential phytohormone pathways. Our findings indicate that GA promotes tuber growth, in part, by inducing *DoDELLA-GAI2* degradation, which subsequently impacts *DoMTCPB* and enhances ETH biosynthesis. This *DoDELLA-GAI2*-*DoMTCPB* interaction represents a crucial regulatory module in the complex phytohormone network governing tuber development in yam. These findings advance our understanding of phytohormone crosstalk and provide a feasible strategy for future molecular breeding.

## Figures and Tables

**Table 1:** BLAST analysis of candidate proteins that interacted with DoDELLA-GAI2 in the yeast two-hybrid system.

ID No.	Functional Comments	Number of Clones	NCBI Blast Serial Number	Comparison Rate/%
Y2H-3	1,2-dihydroxy-3-keto-5-methylthiopentene dioxygenase (MTCPB)	1	XM_039258858	97
Y2H-4	Low-temperature-induced cysteine proteinase	1	XM_020237188	79
Y2H-5	Heme oxygenase 1	1	XM_039286673.1	97
Y2H-7	Histone H2A	1	XM_039280286	91
Y2H-14	Protein DEFECTIVE IN EXINE FORMATION (DEX1)	1	XM_039288871	98
Y2H-15	Triosephosphate isomerase	1	XM_039273555.1	97

## Data Availability

Data are contained within this article.

## Supplementary Materials

The following supporting information can be downloaded at: https://www.mdpi.com/article/10.3390/biology14060635/s1, Figure S1: OPLS-DA and volcano map analysis; Figure S2: Original uncropped agarose gel image for the PCR amplification of the DoDELLA-GAI2 open reading frame (ORF); Figure S3: Original uncropped gel image for the colony PCR screening of potential DoDELLA-GAI2 interacting clones; Figure S4: BiFC confirmed the interaction of DoDELLA-GAI2 with DoMTCPB and DoDEX1 in tobacco cells; Table S1: Primer sequences Table S2: Effects of gibberellin (GA) and paclobutrazol (PAC) treatments on anatomical parameters of yam (Dioscorea opposita) tubers; Table S3: Detected plant hormone information; Table S4: Analysis of metabolic pathways involved in differentially expressed genes and differentially accumulated metabolites; Table S5: Phytohormone signal transduction gene information.

## Abbreviations

The following abbreviations are used in this manuscript:

ABA	Abscisic acid
ACC	1-aminocyclopropane-1-carboxylic acid
BiFC	Bimolecular fluorescence complementation
Cb	Cork cambium
CTK	Cytokinin
Co	Cortex
DAP	Days after planting
DoMTCPB	1,2-dihydroxy-3-keto-5-methylthiopentene dioxygenase
DAMs	Differentially accumulated metabolites
DEGs	Differentially expressed genes
ETH	Ethylene
GA	Gibberellin
Gt	Basic tissue
JA	Jasmonic acid
KEGG	Kyoto Encyclopedia of Genes and Genomes
OPLS-DA	Orthogonal partial least squares discriminant analysis
PAC	Gibberellin biosynthesis inhibitor
PCA	Principal components analysis
Pd	Phelloderm
Pg	Phellem layer
Pi	Pith
SA	Salicylic acid
SSS	Soluble starch synthase
ST	Sieve tube
Ste	Stele
VB	Vascular bundle
VES	Vessel
