# Supplementary material for: DoDELLA-GAI2 Integrates Gibberellin and Ethylene Signaling to Regulate Chinese Yam (Dioscorea opposita) Tuber Development
Source: Biology (Basel). 2025 May 30;14(6):635. doi: 10.3390/biology14060635 (PMC12189092; doi:10.3390/biology14060635)
Supplement: Supplementary file 1 [file biology-14-00635-s001.zip › biology-3607348-supplementary.pdf]

Supplementary Material

DoDELLA-GAI2 Integrates Gibberellin and Ethylene Signaling to Regulate Chinese yam (*Dioscorea opposita*) Tuber Development

Mingran Ge[1], Yanfang Zhang[1], Yanping Xing[2], Linan Xing[1], Huiqin Miao[3] and Xiuwen Huo[1,*]

[1] Horticulture Department, Faculty of Horticulture and Plant Protection Science, Inner Mongolia Agricultural University, Hohhot 010019, China; gemingran1996@163.com (M.G.), zhangyanfang@imau.edu.cn (Y.Z.), xln620719@163.com (L.X.).
[2] Biochemistry and Molecular Biology, School of Life Sciences, Inner Mongolia Agricultural University, Hohhot 010019, China; xyping8315@163.com (Y.X.).
[3] Library, Inner Mongolia Agricultural University, Hohhot 010019, China; miaohuiqin_2010@163.com (H.M.).
* Correspondence: huoxiuwen@imau.edu.cn; Tel.: +86-15184778999

Table S1. Primer sequences.

Primer type	Primer name	Prime sequence (5′→3′)
ORF	DoDELLA-GAI2-ORF-F	ATGGTTCCAAACCCGTTTGG
	DoDELLA-GAI2-ORF-R	ATGCATATACTCATGGAGTG
Transient expression vector	DoDELLA-GAI2--SF	GGTACCATGGTTCCAAACCCGTTTGG
	DoDELLA-GAI2--SR	CCTAGGATGCATATACTCATGGAGTG
	DoMTCBP-SF	GGTACCATGGATGACAGTGAGGAAGACC
	DoMTCBP-SR	GCTCTAGAGTTAGCCTCCACAGCATGACTA
	DoDEX1-SF	GGTACCATGAATCAGGTTTTATTGGTT
	DoDEX1-SR	GCTCTAGAGTAGCTTATAGTAATGCATAT
Plant expression vector	DoDELLA-GAI2--ZF	CCTAGGATGGTTCCAAACCCGTTTGG
	DoDELLA-GAI2--ZR	GGGGTACCCCATGCATATACTCATGGAGTG
Yeast two-hybrid assay	DoDELLA-GAI2-SZ-F	GTCGACAAATGGTTCCAAACCCGTTTGG
	DoDELLA-GAI2-SZ-R	GCTGCAGCATGCATATACTCATGGAGTGTAG
	5'AD	CTATTCGATGATGAAGATACCCC
	3'AD	GTGAACTTGCGGGGTTTTTCAG
AD vector	DoMTCBP-AD-F	GGATCCGCATGGATGACAGTGAGGAAGACC
	DoMTCBP-AD-R	CCCTCGAGGTTAGCCTCCACAGCATGACTATAA
	DoDEX1-AD-F	GGATCCGCATGAATCAGGTTTTATTGGTT
	DoDEX1-AD-R	CCCTCGAGGTAGCTTATAGTAATGCATATTGA
BiFC	DoDELLA-GAI2--CE-F	CTCGAGCATGGTTCCAAACCCGTTTGG
	DoDELLA-GAI2--CE-R	GGGGTACCCCATGCATATACTCATGGAGTG
	DoMTCBP-NE-F	GAATTCGATGGATGACAGTGAGGAAGACC
	DoMTCBP-NE-R	CCCTCGAGGTTAGCCTCCACAGCATGACTA
	DoDEX1-NE-F	CTCGAGCATGAATCAGGTTTTATTGGTT
	DoDEX1-NE-R	GGGGTACCGTAGCTTATAGTAATGCATAT
UBQ	UBQ-F	GGGCTTTCAAGGTCGTC
	UBQ-R	TGAAGGGTTTGCTCATCC

Primer type	Primer name	Prime sequence (5′→3′)
qRT-PCR	DoDELLA-GAI2--qF	AGCACCAAAACAGAGAGAGC
	DoDELLA-GAI2--qR	GAGTCAACCCAAGCGGAGAT
	DoDELLA-GAI1--qF	GGTTGGAGCGTCTGGAGTCTG
	DoDELLA-GAI1--qR	GACATCAGGAGGTGGACGAGT
	DoDELLA-GAI3--qF	GAATGGAGGAGGAGGAGGAC
	DoDELLA-GAI3--qR	GGAGGAAACAGAGTAGAGGC
	DoDELLA-GAI4--qF	GAGGAAGCGGCGGAAGGTGA
	DoDELLA-GAI4--qR	GTGGAGGCAGTGGTTGAGGG
	DoDELLA-SLR1--qF	GTCTTTGGCTTTGTAGTTCCC
	DoDELLA-SLR1--qR	CCATCATCATCTTCCTCCTTG
	DoDELLA-SLR2--qF	CTTTGGCTTTGTAGTTCCCC
	DoDELLA-SLR2--qR	ACTCCATCATCATCTTCCGC
	DoGID1-1--qF	AGTAACGAGGTCAACGCTAA
	DoGID1-1--qR	CGGTAAATACGAACAAGGAG
	DoGID1-2--qF	AAGAAAAAAGACATGGAAGA
	DoGID1-2--qR	CAAGCAATAAAATCAAAACC
	DoGID1-3--qF	CTTTTCTTGTTTAGTGATGG
	DoGID1-3--qR	TTGATAGGAGTAGAGTTTGC
	DoGA2ox-1-qF	TGCCGCTGACTGGGGCTTCT
	DoGA2ox-1-qR	TGTTTCCCTGGCGATTTGTT
	DoGA2ox-2-qF	CCGGCTGGAGAACCATGGGA
	DoGA2ox-2-qR	TCAACGCTGAAAAAGAGGAA
	DoKAO--qF	TTCTCGAGAAAGCCTGATG
	DoKAO--qR	CCACAACAGCCACTGATGA
	DoKS1--qF	CCCTCAACTACTGCTGCTG
	DoKS1--qR	AATTCCTCAACCTTCTGCA
	DoKS2--qF	CATGGCCAACTATGCATGA
	DoKS2--qR	AGGTATCCTTCCATTGAAT
	DoGA20ox--qF	TCACATGCCTTCTTGTTTCA
	DoGA20ox--qR	GGTGTTGGCTTGTCTTCTTC
	DoGA3ox--qF	CACTATCCTCATCTTCCTCT
	DoGA3ox--qR	ACTAACAAACTCACCACCTC
	DoGID2--qF	TCATCGAAGAAGGCAAAGCT
	DoGID2--qR	CAAACACCAGATCATCCCCC
	DoMTCPB--qF	AGACCAGAGGTTTCCACATC
	DoMTCPB--qR	CCGCTCCCTTCAAGACAATA

Table S2. Effects of gibberellin (GA) and paclobutrazol (PAC) treatments on anatomical parameters of yam (*Dioscorea opposita*) tubers.

Sampling time (d)	Treat-ment	Sieve tube diameter (μm)	Number of Sieve tube	Vessel diamete (μm)	Number of vessel	Bundle number	Cork layer thickness	Cork cambium thickness	phelloderm thickness
S1	Con	78.21 ± 12.5ab	18 ± 2.03c	66.67 ± 2.53a	2 ± 0.35a	160 ± 4.18c	129.78 ± 11.43b	249.37 ± 9.7a	105.17 ± 4.6ab
	GA	93.03 ± 5.79a	25 ± 2.03a	214.59 ± 77.1a	2 ± 0.14a	180 ± 3.31a	189.98 ± 17.3a	256.9 ± 9.5a	113.09 ± 4.8a
	PAC	77.31 ± 5.6b	22 ± 2.00b	154.44 ± 17.2a	2 ± 0.23a	176 ± 5.76b	39.38 ± 3.1c	235.9 ± 21a	98.04 ± 0.8b

Notes: Values are presented as mean ± standard deviation (n=3 biological replicates). Con (Control, water spray), GA (200 mg/L gibberellin spray), PAC (200 mg/L paclobutrazol spray). Different lowercase letters (a, b, c) within the same column indicate significant differences between treatments ($P < 0.05$).

Table S3. Detected plant hormone information.

number	English name of compound	Abbreviation	Classification of substances
1	3-Indolebutyric acid	IBA	Auxin
2	Indole-3-carboxaldehyde	ICA	Auxin
3	trans-Zeatin-riboside	tZR	Cytokinin
4	Methyl jasmonate	MeJA	Jasmonic acid
5	N-Jasimonic acid isoleucine	JA-Ile	Jasmonic acid
6	1-Aminocyclopropanecarboxylic acid	ACC	Ethene
7	(±)-Jasmonic acid	JA	Jasmonic acid
8	Salicylic acid	SA	Salicylic acid
9	Abscisic acid	ABA	Abscisic acid
10	GibberellinA3	GA3	Gibberellin
11	GibberellinA4	GA4	Gibberellin

Table S4. Analysis of metabolic pathways involved in differentially expressed genes and differentially accumulated metabolites.

KEGG secondary classification	Metabolic pathway	D_105 vs. D_135		D_135 vs. D_165		D_165 vs. D_105	
		Gene	Metabolite	Gene	Metabolite	Gene	Metabolite
Signal transduction	Plant hormone signal transduction	2	2	-	1	3	2
Metabolism of terpenoids and polyketides	Carotenoid biosynthesis	-	2	-	-	5	1

Table S5. Phytohormone signal transduction gene information.

Phytohormone	Function abbreviation	Function full name
Auxin	AUX1	auxin influx carrier (AUX1 LAX family)
	TIR1	transport inhibitor response 1
	IAA	auxin-responsive protein IAA
	ARF	auxin response factor
	GH3	auxin responsive GH3 gene family
	SAUR	SAUR family protein
Gibberellin	DELLA	DELLA protein
	GID1	gibberellin receptor GID1
	GID2	F-box protein GID2
Cytokinine	CRE1	arabidopsis histidine kinase 2/3/4 (cytokinin receptor)
	AHP	histidine-containing phosphotransfer protein
	B-ARR	two-component response regulator ARR-B family
	A-ARR	two-component response regulator ARR-A family
Abscisic acid	PYR/PYL	abscisic acid receptor PYR/PYL family
	PP2C	protein phosphatase 2C
	SnRK2	serine/threonine-protein kinase SRK2
	ABF	ABA responsive element binding factor
Ethylene	ETR	ethylene receptor
	CTR1	serine/threonine-protein kinase CTR1
	SIMKK	mitogen-activated protein kinase kinase 4/5
	MPK6	mitogen-activated protein kinase 6
	EIN2	ethylene-insensitive protein 2
	EIN3	ethylene-insensitive protein 3
	EBF1_2	EIN3-binding F-box protein
Brassinosteroid	BAK1	brassinosteroid insensitive 1-associated receptor kinase 1
	BRI1	protein brassinosteroid insensitive 1
	BSK	BR-signaling kinase
	BIN2	protein brassinosteroid insensitive 2
	BZR1/2	brassinosteroid resistant 1/2
	TCH4	xyloglucosyl transferase TCH4
Jasmonic acid	JAR1	jasmonic acid-amino synthetase
	COI1	coronatine-insensitive protein 1
	JAZ	jasmonate ZIM domain-containing protein
	MYC2	transcription factor MYC2
Salicylic acid	NPR1	regulatory protein NPR1
	TGA	transcription factor TGA

Figure S1

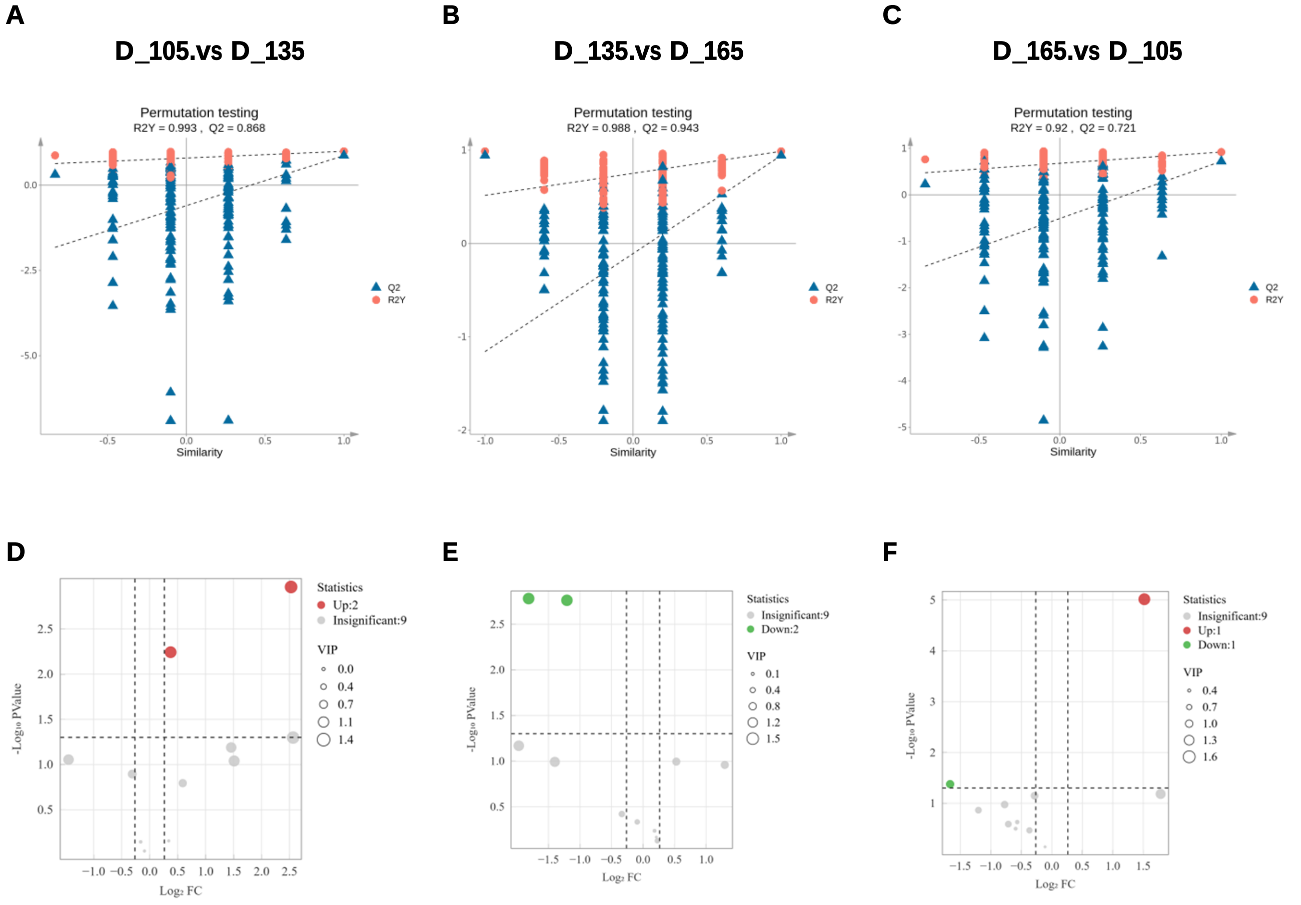

Supplementary Figure S1. OPLS-DA and volcano map analysis.

Note: (A-C): PLS-DA sorting test of yam tuber samples; The horizontal coordinates represent the correlation between the randomly grouped Y and the original grouped Y. The vertical coordinates represent the scores of R2 and Q2; one point in the figure indicates one test. (D-E): Volcano map.

Figure S2

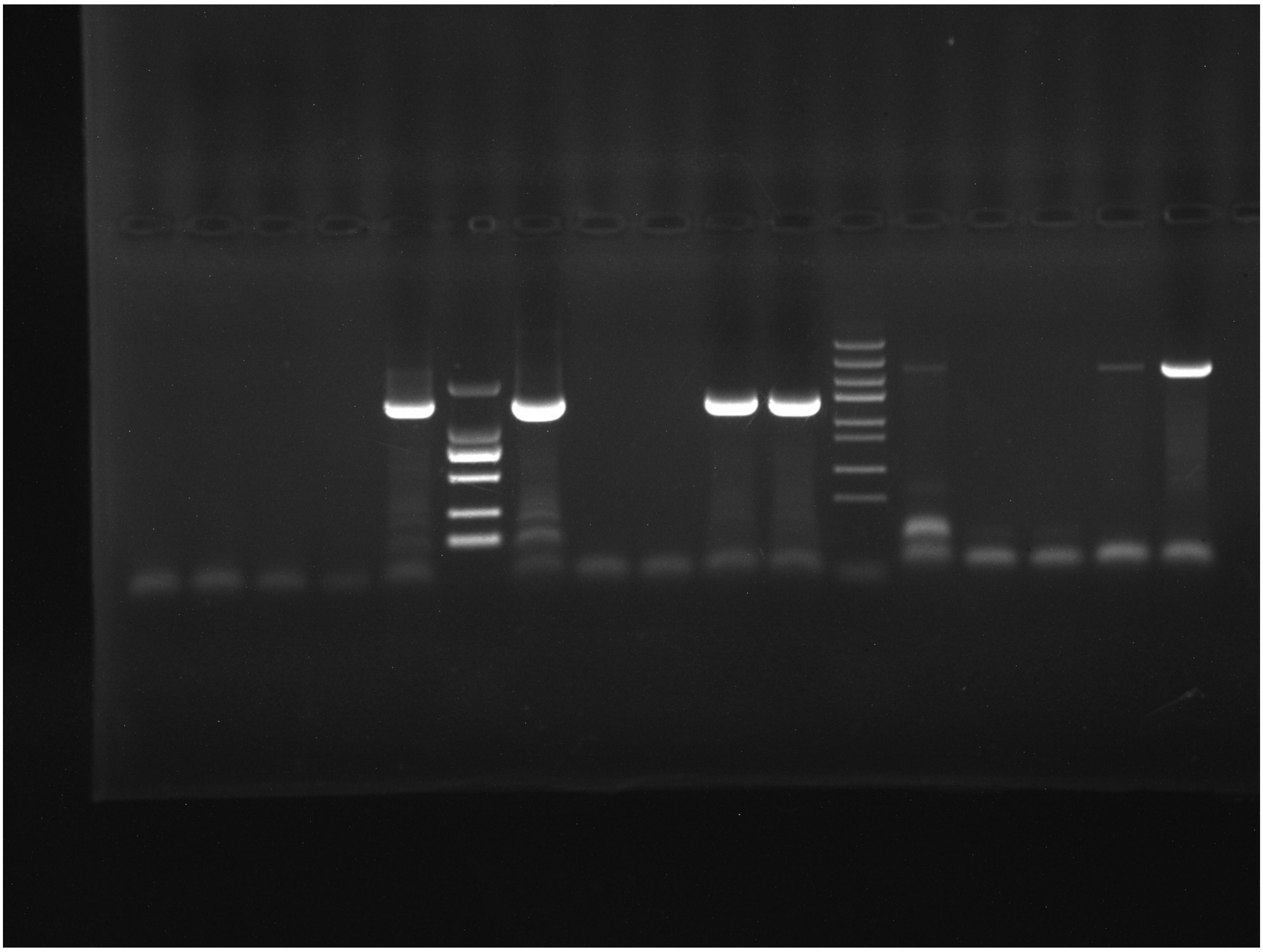

Supplementary Figure S2. Original uncropped agarose gel image for the PCR amplification of the DoDELLA-GAI2 open reading frame (ORF).

Lane 6 from the left: DL2000 DNA Marker; Lane 7: the obtained 1407 bp PCR product.

Figure S3

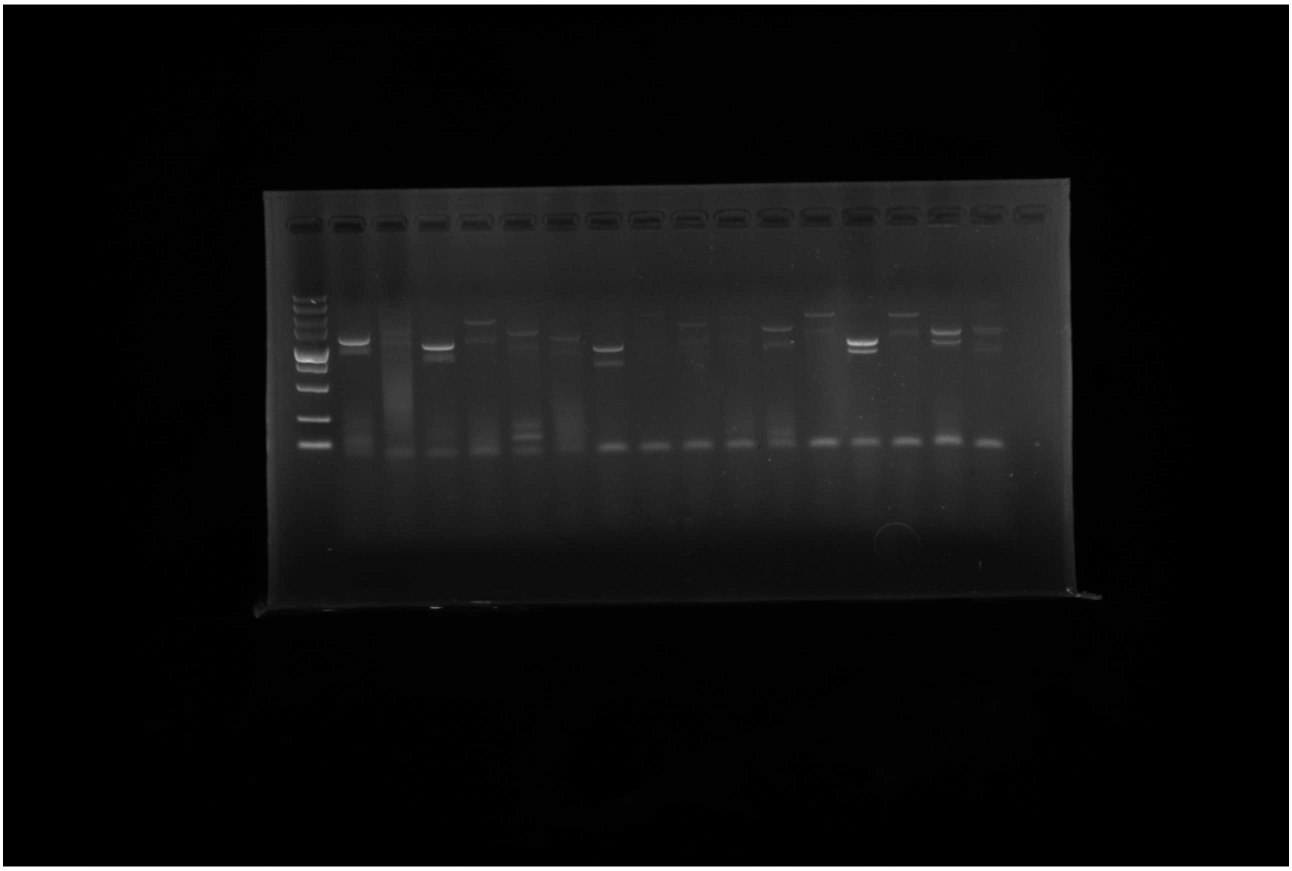

Supplementary Figure S3. Original uncropped gel image for the colony PCR screening of potential DoDELLA-GAI2 interacting clones.

Lane 1 from left: DL5000 DNA marker; Lanes 2-17: PCR products of individual clones (1-16 bacterial solution).

Figure S4

A

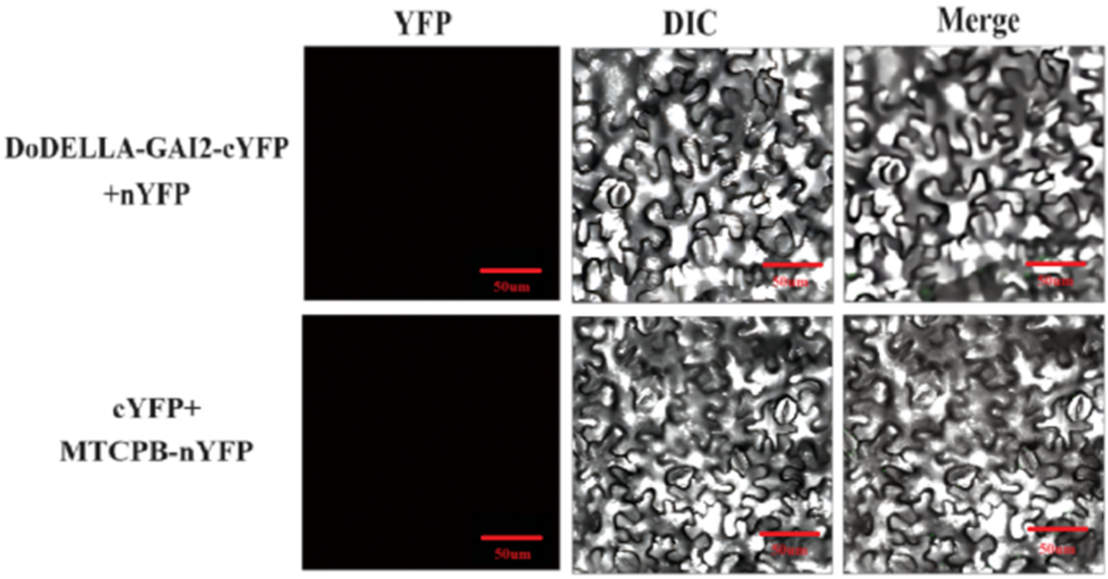

B

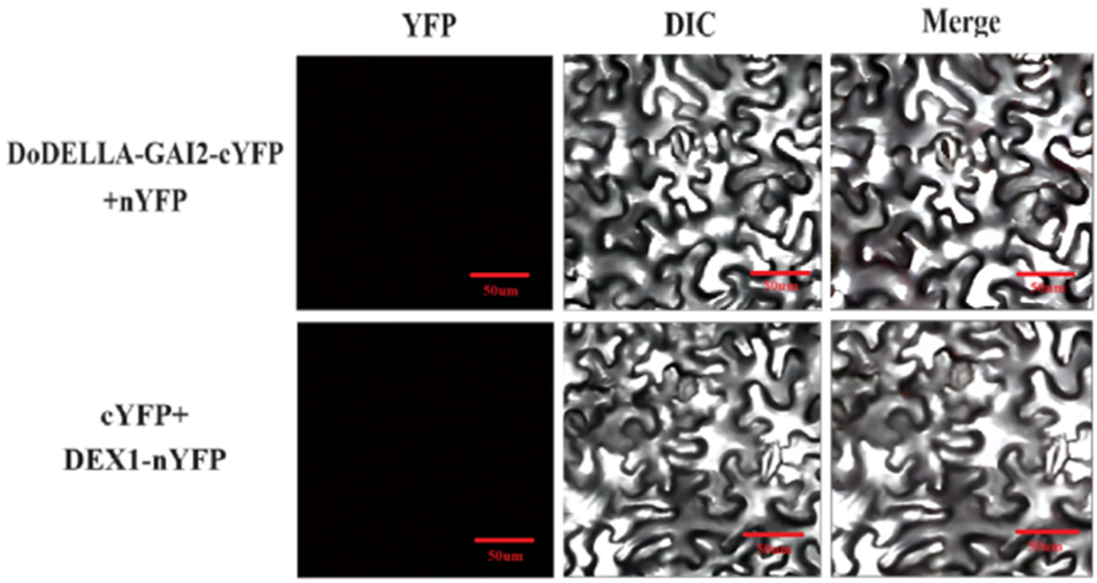

Supplementary Figure S4. BiFC confirmed the interaction of DoDELLA-GAI2 with DoMTCPB and DoDEX1 in tobacco cells. Scale bars: 50 µm.
